# National trends in HIV pre‐exposure prophylaxis awareness, willingness and use among United States men who have sex with men recruited online, 2013 through 2017

**DOI:** 10.1002/jia2.25461

**Published:** 2020-03-09

**Authors:** Patrick S Sullivan, Travis H Sanchez, Maria Zlotorzynska, Cristian J Chandler, RC Sineath, Erin Kahle, Stephen Tregear

**Affiliations:** ^1^ Department of Epidemiology Emory University Atlanta GA USA; ^2^ Department of Health Behavior and Biological Sciences University of Michigan Ann Arbor MI USA; ^3^ Atlas Research Washington DC USA

**Keywords:** PrEP, men who have sex with men, behavioural surveillance, health disparities, PrEP willingness, online surveys, trend analysis, biomedical prevention

## Abstract

**Introduction:**

Pre‐exposure prophylaxis (PrEP) is a key HIV prevention technology, and is a pillar of a comprehensive HIV prevention approach for men who have sex with men (MSM). Because there have been no national data to characterize trends in the PrEP continuum in the United States, overall and for key demographic groups of MSM, we aimed to describe the extent to which PrEP awareness, willingness and use changed over time, overall and for specific groups of MSM critical for HIV prevention (e.g. Black and Hispanic MSM, younger MSM, MSM in rural areas and MSM without health coverage).

**Methods:**

The American Men's Internet Survey (AMIS) is an annual survey of US MSM conducted in the United States among MSM aged ≥15 years since 2013. We analysed data on trends in elements of the PrEP continuum (awareness, willingness and use of PrEP) in a sample of 37,476 HIV‐negative/unknown status MSM from December 2013 through November 2017. We evaluated trends in continuum steps overall and among demographic subgroups using Poisson models with Generalized Estimating Equations. For 2017 data, we used logistic regression to compare the prevalence of PrEP use among demographic groups.

**Results:**

Overall, 51.4% (n = 19,244) of AMIS respondents were PrEP‐eligible across study years. Between 2013 and 2017, PrEP awareness increased from 47.4% to 80.6% willingness to use PrEP increased from 43.9% to 59.5% and PrEP use in the past 12 months increased from 1.7% to 19.9%. In 2017, use of PrEP was lower for men who were younger, lived outside of urban areas, and lacked health insurance; PrEP use was not different among Black, Hispanic and white MSM.

**Conclusions:**

Our data show progress in use of PrEP among US MSM, but also reveal mismatches between PrEP use and epidemic need. We call for additional support of PrEP initiation, especially among young, non‐urban and uninsured MSM. Black and Hispanic MSM report levels of PrEP use no different from white MSM, but given higher HIV incidence for Black and Hispanic MSM, parity in use is not sufficient for epidemic control or health equity.

## 1 Introduction

Men who have sex with men (MSM) are disproportionately impacted by the HIV epidemic in the United States, accounting for about 2% of the US population 1 but two‐thirds of new HIV diagnoses in 2015. Prevention of HIV among MSM will rely on a multicomponent HIV prevention package, including HIV and STI testing, condom promotion, prompt treatment of people living with HIV, and, for those MSM at highest risk, pre‐exposure prophylaxis (PrEP) with antiretroviral medications 2. PrEP can reduce the risk of acquiring HIV infection by 90% among MSM who take it regularly 3. Modelling evidence from the US, South Africa, Peru and India suggests that, to achieve meaningful reductions in new HIV incidence among MSM, PrEP coverage would need to reach 30% to 50% of MSM who meet behavioural eligibility criteria 4, 5, 6. Goals to increase PrEP usage in the United States are part of the US National HIV/AIDS Strategy 7 and the US Department of Health and Human Services plan for Ending the HIV Epidemic in the United States calls for increasing coverage of PrEP for at‐risk Americans 8.

The challenge of achieving high levels of PrEP use among MSM has been conceptualized as a continuum, with milestones of PrEP awareness, willingness to take PrEP, clinical evaluation for PrEP, being PrEP eligible, starting PrEP and persisting on PrEP while risk persists. Understanding the programmatic challenges to improving PrEP use relies on developing data about these phases of PrEP use which are robust, which reflect geographically and racially diverse populations of MSM, and which are collected with consistent methods over time. Measuring PrEP indicators among online samples of MSM offers many desirable features, including broad geographic scope, the inclusion of rural MSM, flexibility and timeliness.

In this study, our objective was to describe key indicators of the PrEP continuum (e.g. PrEP awareness, willingness and use) among MSM using data from the American Men's Internet Survey (AMIS), a nationwide annual online survey that has been functioning continuously since 2013. We also aimed to describe the extent to which the indicators and their change over time were different in specific groups of MSM critical for HIV prevention (e.g. Black and Hispanic MSM, younger MSM, MSM in rural areas and MSM without health coverage).

## 2 Methods

### 2.1 Study population

AMIS is conducted in annual cycles with a goal of obtaining ≥10,000 complete surveys from eligible MSM each cycle. The AMIS methodology has been previously reported 9. Briefly, participants are recruited through convenience sampling from a variety of websites and social media applications using advertisements (hereafter referred to as “ads”). Men who click on ads are taken directly to the survey website hosted on a secure server administered by SurveyGizmo (Boulder, CO, USA). The first page that men encountered on the study website contained a brief description of the study. Those who were interested in participating clicked a "begin survey" button that took them to the study's informed consent page which contained standard information regarding the study purpose, procedures, risks, benefits, protections and investigator contact information. Those who consented to participate in the study were asked to check a box affirming this decision before continuing. The study was conducted in compliance with federal regulations governing protection of human subjects and was reviewed and approved by our institution's human subjects research review board (protocol IRB00047676). Participants from 2015 onward may have also been recruited by emailing participants from the previous cycles of AMIS who consented to be re‐contacted for future studies.

The survey is self‐administered, can be taken on a computer or mobile device and includes questions on demographics, sexual behaviours, substance use, HIV and sexually transmitted infection (STI) testing and diagnosis, and use of HIV prevention services (see online supplemental material for full AMIS‐2017 survey 10). The following AMIS data collection cycles were used for this study: December 2013‐May 2014 (AMIS‐2013), October 2014‐April 2015 (AMIS‐2014), September 2015‐April 2016 (AMIS‐2015), September 2016‐February 2017 (AMIS‐2016) and July through November 2017 (AMIS‐2017).

Participants were eligible for AMIS if they reported being assigned male sex at birth, resided in the US and engaged in oral or anal sex with a man at least once in the past. In AMIS‐2013, only participants aged ≥18 years were eligible. For AMIS‐2014 onward, participants aged ≥15 years were eligible. Participants who met the eligibility criteria and consented to participate in the study started the online survey immediately. No incentives were provided to participants. To illustrate the characteristics of 2017 AMIS respondents compared to the US population in 2017, we compared AMIS participants to population‐based data sources with respect to race/ethnicity, Census region of residence, urbanicity and health insurance status (Figure S1).

### 2.2 Measures

We examined three outcome measures: PrEP awareness, willingness to use PrEP, and usage of PrEP in the past 12 months. Only participants who did not report having been previously diagnosed with HIV infection (i.e. whose last HIV test was negative, or who were never tested for HIV) were asked the PrEP questions. These men were provided with a brief description of PrEP and then were asked about their awareness of PrEP “before today” (see supplemental material for full set of PrEP questions 10). Willingness to use PrEP was assessed with the question, “Would you be willing to take anti‐HIV medicines every day to lower your chances of getting HIV?”. Use of PrEP was assessed with the question, “In the past 12 months (since [MONTH/YEAR]), have you taken PrEP?” Participants who did not report using PrEP within the past 12 months were given details about PrEP and then were asked about their willingness to use it. In AMIS‐2014, there was a question logic error that did not show the willingness question for some participants thus these data are not available for every eligible participant in that year. Participants who had been on PrEP in the past 12 months were not asked questions about willingness.

We determined PrEP eligibility for participants using an algorithm based on CDC guidelines matched as closely as possible to AMIS questions 11. To be considered eligible for PrEP, participants age 18 and older must have reported more than one male sexual partner in the past 12 months and also reported at least one of the following: anal sex with a man without a condom in the past 12 months; diagnosis of gonorrhoea, chlamydia or syphilis in the past 12 months; or had a main male partner who was living with HIV infection. For the last criterion, CDC identifies any ongoing sexual relationship with an HIV‐positive male partner as a risk factor, but we did not ask about duration or continuation of sexual partnerships and used main partner as a proxy.

Independent measures included age, race/ethnicity, residency, health insurance coverage, and recruitment source. Participant‐provided ZIP codes were used to determine region of residency and residence in a city included in the CDC National HIV Behavioral Surveillance System (NHBS) 12. Participant's residential population density was assessed at the county‐level using the National Center for Health Statistics (NCHS) Rural‐Urban classification scheme 13. We further collapsed these categories into a four‐level population density variable: urban (central), suburban (fringe), medium/small metropolitan and rural (micropolitan and non‐core). Current health insurance coverage was self‐reported and participants were categorized as having either no insurance, private insurance only, public insurance only (e.g. Medicare, Medicaid, Veterans Administration), or other/multiple types of insurance.

Banner ads and email blasts contained unique links, which allowed us to determine from which website or app participants entered the survey. We categorized these by target audience and purpose: gay social networking, gay general interest, general social networking and geospatial social networking (i.e. “dating” apps). Some participants completed the survey during previous data collection cycles; participants recruited from previous AMIS cycles were categorized according to their original recruitment source for analyses of behaviours.

### 2.3 Statistical analyses

Eligible consenting AMIS participants were included in the present analyses if they were unduplicated by IP address within the survey year, completed the survey, had sex with a man in the past 12 months, provided a valid US ZIP code, were age 18 and older, and did not report being HIV positive. Methods and results for these recruitment and enrolment analytics have been previously reported 9. Overall, chi‐square tests were used to assess whether participant characteristics differed significantly among annual recruitment cycles for PrEP eligibility of participants. Trend in PrEP eligibility was assessed by the Cochrane Armitage test; overall and excluding 2013. All other analyses included participants who were eligible for PrEP and we report proportions and 95% confidence intervals (CI) or prevalence ratios and 95% CI.

Cochran‐Armitage tests for trend and Poisson models using Generalized Estimating Equations (GEE) were used to test for a linear trend between AMIS‐2013, AMIS‐2014, AMIS‐2015, AMIS‐2016 and AMIS‐2017 for PrEP awareness, willingness and usage in the past 12 months, overall and among demographic sub‐groups of participants. All GEE trend models included the following covariates: age, race/ethnicity, recruitment source, population density and health insurance coverage. AMIS cycle and age were treated as continuous variables in modelling. Models included interaction terms for AMIS cycle by age as appropriate. For models where the age by AMIS cycle interaction term was significant, model findings stratified by age group are presented. No significant interactions with other model variables were found (data not presented).

Trends in PrEP measures by participant race/ethnicity (non‐Hispanic white and other) were plotted. Because previous AMIS analyses have identified consistent statistically significant associations between other behaviours and recruitment source, the prevalence of each PrEP measure is adjusted for recruitment source, using AMIS‐2017 as the standard population 9. Due to the adjustment, we were unable to use the GEE modelling and instead calculated estimated annual percentage change (EAPC) with 95% CI which has been previously used to examine trends in behavioural surveillance indicators 14.

To determine which participant characteristics were independently associated with PrEP measures in the most recent cycle (AMIS‐2017), a multivariable logistic regression model was built also using age, race/ethnicity, recruitment source, population density and health insurance coverage. Results are presented as adjusted prevalence ratios (aPR) with 95% CIs.

## 3 Results

There were 37,476 AMIS‐eligible MSM participants in five annual cycles of AMIS that were conducted from December 2013 through November 2017 (Table 1). Most participants in all cycles were age 30 years or older, non‐Hispanic white, and were recruited from general social networking sites. Participants were recruited from all US states (data not presented) and the most common region of residence was the South. Approximately 40% of participants in each AMIS cycle reported residence in an NHBS city. Most participants reported residence in an urban or suburban county. Around one in 10 participants were uninsured and most of those who were insured had private health insurance. Less than 10% of all cycles' samples were comprised of repeat participants.

**Table 1:** Characteristics of MSM participants in the American Men's Internet Survey by survey cycle and eligiblity for HIV pre‐exposure prophylaxis, United States

Participant characteristics	AMIS‐2013^a^		AMIS‐2014^b^		AMIS‐2015^c^		AMIS‐2016^d^		AMIS‐2017^e^	
	Total	PrEP eligible^f^	Total	PrEP eligible^f^	Total	PrEP eligible^f^	Total	PrEP eligible^f^	Total	PrEP eligible^f^
	N (%)	n (%)	N (%)	n (%)	N (%)	n (%)	N (%)	n (%)	N (%)	n (%)
Total	3072 (1165.0)	1907 (62.1)	8045 (3963.0)	4082 (50.7)	9006 (4493.0)	4513 (50.1)	8712 (4445.0)	4267 (49.0)	8641 (4166.0)	4475 (51.8)
Age (years)										
18 to 24	628 (20.4)	411 (21.6)	1223 (15.2)	658 (16.1)	2515 (27.9)	1280 (28.4)	2303 (26.4)	1172 (27.5)	2243 (26.0)	1194 (26.7)
25 to 29	461 (15.0)	318 (16.7)	1098 (13.6)	627 (15.4)	1476 (16.4)	838 (18.6)	1596 (18.3)	902 (21.1)	1168 (13.5)	667 (14.9)
30 to 39	558 (18.2)	393 (20.6)	1694 (21.1)	966 (23.7)	1306 (14.5)	701 (15.5)	1228 (14.1)	645 (15.1)	1420 (16.4)	822 (18.4)
40 and older	1425 (46.4)	785 (41.2)	4030 (50.1)	1831 (44.9)	3709 (41.2)	1694 (37.5)	3585 (41.2)	1548 (36.3)	3810 (44.1)	1792 (40.0)
Race/ethnicity										
Black, non‐Hispanic	99 (3.2)	60 (3.1)	317 (3.9)	156 (3.8)	509 (5.7)	239 (5.3)	611 (7.0)	294 (6.9)	496 (5.7)	264 (5.9)
Hispanic	301 (9.8)	198 (10.4)	1096 (13.6)	602 (14.7)	1185 (13.2)	615 (13.6)	1120 (12.9)	589 (13.8)	1255 (14.5)	675 (15.1)
White, non‐Hispanic	2417 (78.7)	1494 (78.3)	6011 (74.7)	3003 (73.6)	6547 (72.7)	3280 (72.7)	6214 (71.3)	3018 (70.7)	6109 (70.7)	3161 (70.6)
Other or multiple races	255 (8.3)	155 (8.1)	621 (7.7)	321 (7.9)	765 (8.5)	379 (8.4)	767 (8.8)	366 (8.6)	591 (6.8)	304 (6.8)
Recruitment type										
Gay social networking	274 (8.9)	143 (7.5)	353 (4.4)	167 (4.1)	1341 (14.9)	613 (13.6)	1035 (11.9)	457 (10.7)	1148 (13.3)	514 (11.5)
General gay interest	577 (18.8)	356 (18.7)	335 (4.2)	171 (4.2)	353 (3.9)	172 (3.8)	11 (0.1)	4 (0.1)	73 (0.8)	31 (0.7)
General social networking	1638 (53.3)	994 (52.1)	5327 (66.2)	2483 (60.8)	4735 (52.6)	2322 (51.5)	5432 (62.4)	2583 (60.5)	2954 (34.2)	1523 (34.0)
Geospatial social networking	583 (19.0)	414 (21.7)	2030 (25.2)	1261 (30.9)	2478 (27.5)	1350 (29.9)	1482 (17.0)	783 (18.4)	3480 (40.3)	1809 (40.4)
Previous year's participants	–		–	–	99 (1.1)	56 (1.2)	752 (8.6)	440 (10.3)	986 (11.4)	598 (13.4)
Region										
Northeast	663 (21.6)	392 (20.6)	1381 (17.2)	681 (16.7)	1828 (20.3)	825 (18.3)	1647 (18.9)	810 (19.0)	1635 (18.9)	828 (18.5)
Midwest	612 (19.9)	389 (20.4)	1719 (21.4)	855 (20.9)	1908 (21.2)	1002 (22.2)	1725 (19.8)	812 (19.0)	1657 (19.2)	863 (19.3)
South	1058 (34.4)	655 (34.3)	3139 (39.0)	1572 (38.5)	3244 (36.0)	1630 (36.1)	3434 (39.4)	1716 (40.2)	3286 (38.0)	1675 (37.4)
West	737 (24.0)	469 (24.6)	1797 (22.3)	966 (23.7)	2018 (22.4)	1050 (23.3)	1902 (21.8)	927 (21.7)	2056 (23.8)	1107 (24.7)
U.S. dependent areas	2 (0.1)	2 (0.1)	9 (0.1)	8 (0.2)	8 (0.1)	6 (0.1)	4 (0.0)	2 (0.0)	7 (0.1)	2 (0.0)
NHBS city resident										
Yes	1183 (38.5)	737 (38.6)	3013 (37.5)	1518 (37.2)	3236 (35.9)	1601 (35.5)	3552 (40.8)	1754 (41.1)	3430 (39.7)	1799 (40.2)
No	1889 (61.5)	1170 (61.4)	5032 (62.5)	2564 (62.8)	5770 (64.1)	2912 (64.5)	5160 (59.2)	2513 (58.9)	5211 (60.3)	2676 (59.8)
Population density^g^										
Urban	1349 (43.9)	861 (45.1)	3373 (41.9)	1760 (43.1)	3559 (39.5)	1826 (40.5)	3635 (41.7)	1829 (42.9)	3633 (42.0)	1940 (43.4)
Suburban	598 (19.5)	358 (18.8)	1596 (19.8)	760 (18.6)	1803 (20.0)	851 (18.9)	1880 (21.6)	883 (20.7)	1837 (21.3)	901 (20.1)
Small/medium metropolitan	824 (26.8)	501 (26.3)	2360 (29.3)	1180 (28.9)	2751 (30.5)	1405 (31.1)	2437 (28.0)	1175 (27.5)	2460 (28.5)	1274 (28.5)
Rural	299 (9.7)	185 (9.7)	706 (8.8)	374 (9.2)	882 (9.8)	422 (9.4)	756 (8.7)	378 (8.9)	703 (8.1)	357 (8.0)
Health Insurance										
None	270 (8.8)	176 (9.2)	719 (8.9)	287 (7.0)	968 (10.7)	488 (10.8)	1394 (16.0)	667 (15.6)	765 (8.9)	399 (8.9)
Private only	1653 (53.8)	1040 (54.5)	5686 (70.7)	2969 (72.7)	5959 (66.2)	3009 (66.7)	5359 (61.5)	2723 (63.8)	5887 (68.1)	3143 (70.2)
Public only	176 (5.7)	90 (4.7)	619 (7.7)	331 (8.1)	917 (10.2)	427 (9.5)	886 (10.2)	380 (8.9)	959 (11.1)	452 (10.1)
Other/multiple	254 (8.3)	129 (6.8)	616 (7.7)	288 (7.1)	857 (9.5)	445 (9.9)	766 (8.8)	358 (8.4)	754 (8.7)	358 (8.0)

Chi‐square tests for difference in participant characteristics between AMIS cycles for PrEP eligibility were conducted for all characteristics. All group comparisons were significant at *p* < 0.0001. AMIS, American Men's Internet Survey; MSM, men who sex With Men; NHBS, National HIV Behavioral Surveillance; PrEP, HIV pre‐exposure prophylaxis.

a Data collected between December 2013 and May 2014

b data collected between October 2014 and April 2015

c data collected between September 2015 and April 2016

d data collected between September 2016 and February 2017

e data collected between July 2017 and November 2017

f PrEP eligibility was determined by CDC guidelines for behavioral risk assessment of MSM. Proportion PrEP eligible are among all of those in each participant sub‐group

g there were 10 participants in 2013, 11 in 2014, 11 in 2015, 4 in 2016 and 11 in 2017 who reported living in US territories or provided military addresses, which could not have an NCHS urban/rural category assigned.

Overall, 51.4% (19,244/37,476) of MSM participants in AMIS were also PrEP eligible. There was a decreasing trend in PrEP eligibility by year, but this decreased appeared to be driven by decreases in eligibility between 2013 and 2014. Excluding 2013, PrEP eligibility did not vary significantly by AMIS cycle from 2014 to 2017 (Table 1). Among PrEP‐eligible MSM, there were differences across AMIS cycles in all participant characteristics.

### 3.1 Trends in awareness of PrEP

**Figure 1:**
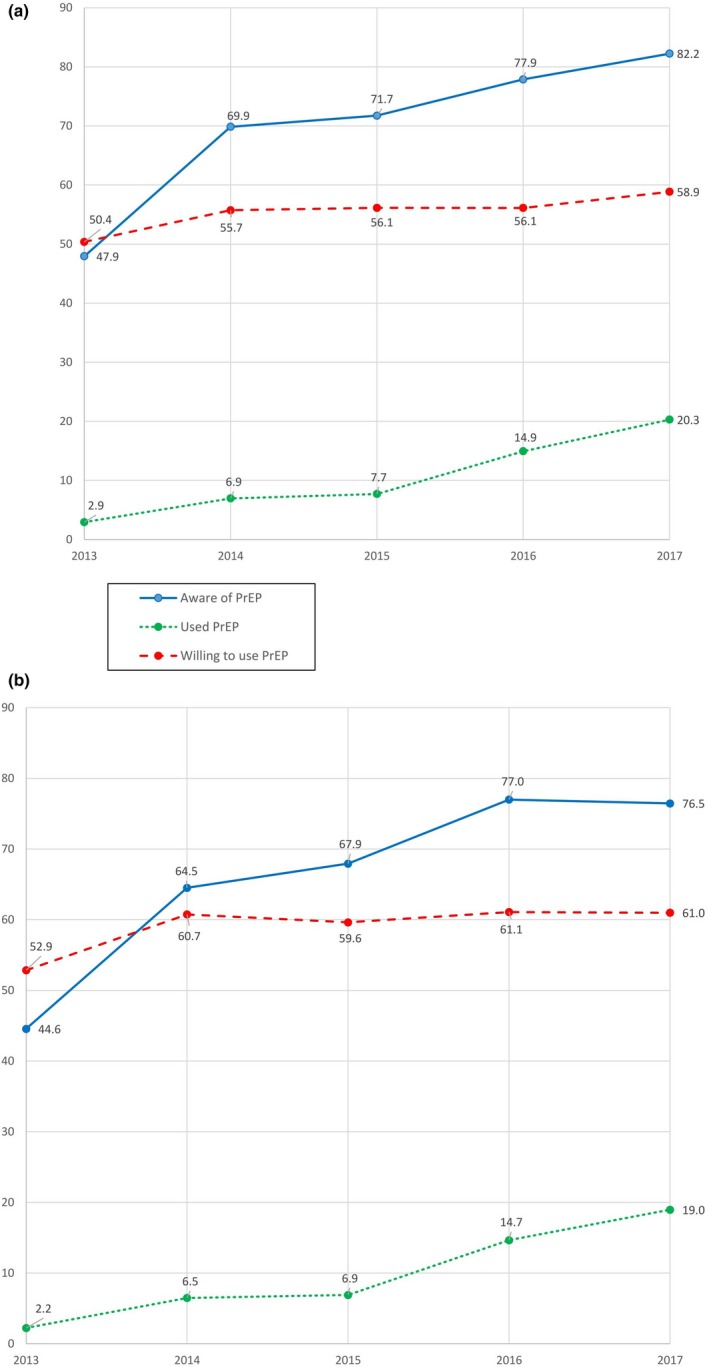
PrEP awareness, willingness and use among (a) non‐Hispanic white MSM (b) men other than non‐Hispanic white MSM, 2013 to 2017, United States.

The proportion of PrEP‐eligible MSM who were aware of PrEP increased from 47.4% in AMIS‐2013 to 80.6% in AMIS‐2017 (Table 2). When standardizing by recruitment type across AMIS cycles, non‐Hispanic white MSM had a +12.6% (Figure 1a) EAPC and MSM of other races/ethnicity had a +13.4% EAPC (Figure 1b), indicating annual increases in PrEP awareness. PrEP awareness significantly increased across all other subgroups analysed (Table 2).

**Table 2:** Awareness of HIV pre‐exposure prophylaxis among PrEP‐eligible MSM participants in the American Men's Internet Survey by survey cycle, United States

	AMIS‐2013^a^	AMIS‐2014^b^	AMIS‐2015^c^	AMIS‐2016^d^	AMIS‐2017^e^	aPR (95% CI) for 2017 sample^f^
	n/N (%)	n/N (%)	n/N (%)	n/N (%)	n/N (%)	
Total	903/1907 (47.4)	2784/4082 (68.2)	3180/4513 (70.5)	3421/4267 (80.2)	3605/4475 (80.6)	
Age (years)						
18 to 24	143/411 (34.8)	341/658 (51.8)	844/1280 (65.9)	883/1172 (75.3)	919/1194 (77.0)	**0.92 (0.88, 0.96)**
25 to 29	165/318 (51.9)	454/627 (72.4)	663/838 (79.1)	777/902 (86.1)	571/667 (85.6)	REF
30 to 39	219/393 (55.7)	707/966 (73.2)	540/701 (77.0)	555/645 (86.0)	709/822 (86.3)	1.01 (0.97, 1.05)
40 and older	376/785 (47.9)	1282/1831 (70.0)	1133/1694 (66.9)	1206/1548 (77.9)	1406/1792 (78.5)	**0.91 (0.87, 0.95)**
Race/ethnicity						
Black, non‐Hispanic	30/60 (50.0)	100/156 (64.1)	176/239 (73.6)	238/294 (81.0)	214/264 (81.1)	0.96 (0.90, 1.02)
Hispanic	87/198 (43.9)	384/602 (63.8)	391/615 (63.6)	458/589 (77.8)	503/675 (74.5)	**0.90 (0.86, 0.95)**
White, non‐Hispanic	719/1494 (48.1)	2091/3003 (69.6)	2344/3280 (71.5)	2447/3018 (81.1)	2600/3161 (82.3)	REF
Other or multiple races	67/155 (43.2)	209/321 (65.1)	269/379 (71.0)	278/366 (76.0)	235/304 (77.3)	**0.92 (0.86, 0.98)**
Recruitment type						
Gay social networking	35/143 (24.5)	86/167 (51.5)	358/613 (58.4)	384/507 (75.7)	461/592 (77.9)	0.96 (0.91, 1.01)
General gay interest	223/356 (62.6)	134/171 (78.4)	152/172 (88.4)	37/41 (90.2)	41/48 (85.4)	0.99 (0.87, 1.13)
General social networking	387/994 (38.9)	1587/2483 (63.9)	1637/2348 (69.7)	2192/2770 (79.1)	1543/1893 (81.5)	REF
Geospatial social networking	258/414 (62.3)	977/1261 (77.5)	1033/1376 (75.1)	796/937 (85.0)	1557/1939 (80.3)	**0.96 (0.93, 0.99)**
Population density^g^						
Urban	477/861 (55.4)	1337/1760 (76.0)	1397/1826 (76.5)	1557/1829 (85.1)	1608/1940 (82.9)	REF
Suburban	157/358 (43.9)	512/760 (67.4)	587/851 (69.0)	706/883 (80.0)	714/901 (79.2)	0.96 (0.92, 1.00)
Small/medium metropolitan	199/501 (39.7)	745/1180 (63.1)	947/1405 (67.4)	913/1175 (77.7)	1024/1274 (80.4)	**0.97 (0.93, 1.00)**
Rural	70/185 (37.8)	185/374 (49.5)	244/422 (57.8)	243/378 (64.3)	257/357 (72.0)	**0.88 (0.82, 0.94)**
Health Insurance						
None	58/176 (33.0)	199/287 (69.3)	299/488 (61.3)	469/667 (70.3)	327/399 (82.0)	REF
Private only	507/1040 (48.8)	2102/2969 (70.8)	2250/3009 (74.8)	2327/2723 (85.5)	2586/3143 (82.3)	1.00 (0.95, 1.05)
Public only	32/90 (35.6)	199/331 (60.1)	254/427 (59.5)	265/380 (69.7)	335/452 (74.1)	**0.92 (0.85, 0.99)**
Other/multiple	53/129 (41.1)	172/288 (59.7)	304/445 (68.3)	268/358 (74.9)	283/358 (79.1)	0.99 (0.93, 1.07)
Region						
Northeast	192/392 (49.0)	476/681 (69.9)	610/825 (73.9)	675/810 (83.3)	657/828 (79.3)	
Midwest	181/389 (46.5)	550/855 (64.3)	683/1002 (68.2)	634/812 (78.1)	715/863 (82.9)	
South	304/655 (46.4)	1063/1572 (67.6)	1141/1630 (70.0)	1368/1716 (79.7)	1337/1675 (79.8)	
West	226/469 (48.2)	690/966 (71.4)	743/1050 (70.8)	742/927 (80.0)	895/1107 (80.8)	
U.S. dependent areas	0/2 (0.0)	5/8 (62.5)	3/6 (50.0)	2/2 (100.0)	1/2 (50.0)	
NHBS city resident						
Yes	426/737 (57.8)	1156/1518 (76.2)	1212/1601 (75.7)	1492/1754 (85.1)	1478/1799 (82.2)	
No	477/1170 (40.8)	1628/2564 (63.5)	1968/2912 (67.6)	1929/2513 (76.8)	2127/2676 (79.5)	

Chi‐square tests for trend across AMIS cycles in PrEP awareness overall and within participant subgroups were significant (*p* < 0.0001) for all characteristics except US. Dependent areas (*p* = 0.35). A GEE model for linear trend across AMIS cycles in PrEP awareness overall, controlling for age, race/ethnicity, recruitment source, population density, and health insurance coverage, indicated a significant trend (*p* < 0.0001) overall and for all age groups. aPR, adjusted prevalence ratios; AMIS, American Men's Internet Survey; GEE, generalized estimating equation; MSM, men who sex with men; NHBS, National HIV Behavioral Surveillance; PrEP, HIV pre‐exposure prophylaxis. Bolded values indicate confidedence intervals that exclude 1.0.

a Data collected between December 2013 and May 2014

b data collected between October 2014 and April 2015

c data collected between September 2015 and April 2016

d data collected between September 2016 and February 2017

e data collected between July 2017 and November 2017

f calculated for 2017 sample, using GEE model that adjusted for age (as a categorical variable), race/ethnicity, recruitment source, population density and health insurance coverage

g there were 10 participants in 2013, 11 in 2014, 11 in 2015, 4 in 2016 and 11 in 2017 who reported living in US territories or provided military addresses, which could not have an NCHS urban/rural category assigned.

### 3.2 Trends in willingness to Use PrEP

The proportion of PrEP‐eligible MSM who were willing to use PrEP increased significantly from 43.9% in AMIS‐2013 to 59.5% in AMIS‐2017 (Table 3). Black non‐Hispanic, white non‐Hispanic and Hispanic MSM all experienced significant increases in willingness to use PrEP over the period. MSM recruited from gay general interest websites, from general social networking websites and from gay social networking had increased willingness, but those recruited from geospatial social networking did not. Several groups of MSM did not experience a significant increase in willingness to use PrEP: MSM residing in rural areas, MSM who had public or other health insurance, and men who reported multiple race/ethnicities or single race/ethnicities other than white non‐Hispanic, Black‐non‐Hispanic or Hispanic.

**Table 3:** Willingness to use HIV pre‐exposure prophylaxis among PrEP‐eligible MSM participants in the American Men's Internet Survey by survey cycle, United States

	AMIS‐2013^a^	AMIS‐2014^b^	AMIS‐2015^c^	AMIS‐2016^d^	AMIS‐2017^e^	aPR (95% CI) for 2017 sample^f^
	n/N (%)	n/N (%)	n/N (%)	n/N (%)	n/N (%)	
Total	822/1874 (43.9)	1520/2746 (55.4)	2367/4167 (56.8)	2180/3695 (59.0)	2132/3584 (59.5)	
Age (years)						
18 to 24	212/406 (52.2)	287/486 (59.1)	791/1220 (64.8)	747/1097 (68.1)	720/1082 (66.5)	**1.13 (1.03, 1.23)**
25 to 29	144/311 (46.3)	246/422 (58.3)	456/761 (59.9)	453/740 (61.2)	305/503 (60.6)	REF
30 to 39	154/382 (40.3)	356/609 (58.5)	360/621 (58.0)	312/524 (59.5)	340/574 (59.2)	0.98 (0.89, 1.08)
40 and older	312/775 (40.3)	631/1229 (51.3)	760/1565 (48.6)	668/1334 (50.1)	767/1425 (53.8)	**0.89 (0.81, 0.97)**
Race/ethnicity						
Black, non‐Hispanic	29/60 (48.3)	50/98 (51.0)	131/226 (58.0)	157/260 (60.4)	128/203 (63.1)	1.05 (0.94, 1.18)
Hispanic	97/195 (49.7)	266/423 (62.9)	336/574 (58.5)	330/508 (65.0)	353/565 (62.5)	1 (0.92, 1.08)
White, non‐Hispanic	622/1469 (42.3)	1068/2000 (53.4)	1684/3018 (55.8)	1499/2604 (57.6)	1482/2519 (58.8)	REF
Other or multiple races	74/150 (49.3)	136/225 (60.4)	216/349 (61.9)	194/323 (60.1)	139/239 (58.2)	0.93 (0.82, 1.04)
Recruitment type						
Gay social networking	59/141 (41.8)	64/121 (52.9)	302/577 (52.3)	244/442 (55.2)	274/477 (57.4)	1.08 (0.98, 1.19)
General gay interest	106/354 (29.9)	67/120 (55.8)	85/155 (54.8)	19/34 (55.9)	24/36 (66.7)	1.09 (0.84, 1.43)
General socialnetworking	409/987 (41.4)	899/1743 (51.6)	1244/2195 (56.7)	1430/2455 (58.2)	943/1573 (59.9)	REF
Geospatial social networking	248/392 (63.3)	490/762 (64.3)	736/1240 (59.4)	477/754 (63.3)	890/1496 (59.5)	1.02 (0.96, 1.08)
Population density^g^						
Urban	367/836 (43.9)	631/1137 (55.5)	917/1633 (56.2)	833/1451 (57.4)	821/1417 (57.9)	REF
Suburban	153/356 (43.0)	284/509 (55.8)	436/800 (54.5)	477/803 (59.4)	438/756 (57.9)	1.02 (0.94, 1.10)
Small/medium metropolitan	219/495 (44.2)	434/813 (53.4)	774/1318 (58.7)	667/1080 (61.8)	684/1088 (62.9)	**1.08 (1.01, 1.15)**
Rural	83/185 (44.9)	164/279 (58.8)	236/407 (58.0)	203/359 (56.5)	187/320 (58.4)	1.02 (0.92, 1.13)
Health insurance						
None	72/178 (40.4)	86/184 (46.7)	303/479 (63.3)	351/605 (58.0)	264/374 (70.6)	REF
Private only	398/1030 (38.6)	1092/1970 (55.4)	1521/2716 (56.0)	1387/2291 (60.5)	1427/2417 (59.0)	**0.86 (0.80, 0.93)**
Public only	41/90 (45.6)	121/229 (52.8)	237/402 (59.0)	194/338 (57.4)	214/372 (57.5)	**0.85 (0.76, 0.95)**
Other/multiple	51/126 (40.5)	115/204 (56.4)	227/426 (53.3)	175/325 (53.8)	162/300 (54.0)	**0.79 (0.69, 0.89)**
Region						
Northeast	145/387 (37.5)	224/462 (48.5)	395/763 (51.8)	368/690 (53.3)	356/653 (54.5)	
Midwest	161/379 (42.5)	319/592 (53.9)	522/929 (56.2)	441/716 (61.6)	435/713 (61.0)	
South	324/648 (50.0)	598/1062 (56.3)	888/1518 (58.5)	888/1494 (59.4)	818/1363 (60.0)	
West	192/458 (41.9)	372/622 (59.8)	559/951 (58.8)	483/793 (60.9)	522/853 (61.2)	
U.S. dependent areas	0 (0.0)	7/8 (87.5)	3/6 (50.0)	0/2 (0.0)	1/2 (50.0)	
NHBS city resident						
Yes	303/719 (42.1)	532/965 (55.1)	794/1434 (55.4)	836/1429 (58.5)	742/1333 (55.7)	
No	519/1155 (44.9)	988/1781 (55.5)	1573/2733 (57.6)	1344/2266 (59.3)	1390/2251 (61.8)	

Chi‐square tests for trend across AMIS cycles in willingness to use overall and within participant subgroups were significant (*p* < 0.05) for all characteristics except geospatial social networking apps, public only insurance, and other/multiple insurance. A GEE model for linear trend across AMIS cycles in PrEP willingness overall, controlling for age, race/ethnicity, recruitment source, population density, and health insurance coverage, indicated a significant trend (*p* < 0.0001) overall. aPR, adjusted prevalence ratios; AMIS, American Men's Internet Survey; GEE, generalized estimating equation; MSM, men who sex with men; NHBS, National HIV Behavioral Surveillance; PrEP, HIV pre‐exposure prophylaxis. Bolded values indicate confidedence intervals that exclude 1.0.

a Data collected between December 2013 and May 2014

b data collected between October 2014 and April 2015

c Data collected between September 2015 and April 2016

d data collected between September 2016 and February 2017

e data collected between July 2017 and November 2017

f calculated for 2017 sample, using GEE model that adjusted for age (as a categorical variable), race/ethnicity, recruitment source, population density and health insurance coverage

g there were 10 participants in 2013, 11 in 2014, 11 in 2015, 4 in 2016 and 11 in 2017 who reported living in US territories or provided military addresses, which could not have an NCHS urban/rural category assigned.

### 3.3 Trends in usage of PrEP

The proportion of PrEP‐eligible MSM who had used PrEP in the past 12 months increased from 1.7% in AMIS‐2013 to 19.9% in AMIS‐2017 (Table 4). Every racial/ethnic group of MSM had a significant increase in PrEP usage between AMIS‐2013 and AMIS‐2017; for Black MSM, the proportion of PrEP‐eligible MSM increased from 0% in 2013 to 23.1% in 2017. When standardizing by recruitment type across AMIS cycles, non‐Hispanic white MSM had a +58.8% (Figure 1a,b) EAPC for PrEP usage and MSM of other races/ethnicities had an annual increase of +66.6% of PrEP usage from AMIS‐2013 to AMIS‐2017 (Table 5).

**Table 4:** Use of HIV pre‐exposure prophylaxis among PrEP‐eligible MSM participants in the American Men's Internet Survey by survey cycle, United States

	AMIS‐2013^a^	AMIS‐2014^b^	AMIS‐2015^c^	AMIS‐2016^d^	AMIS‐2017^e^	aPR (95% CI) for 2017 sample^f^
	n/N (%)	n/N (%)	n/N (%)	n/N (%)	n/N (%)	
Total	33/1907 (1.7)	247/4082 (6.1)	346/4513 (7.7)	572/4267 (13.4)	891/4475 (19.9)	
Age (years)						
18 to 24	5/411 (1.2)	19/658 (2.9)	60/1280 (4.7)	75/1172 (6.4)	112/1194 (9.4)	**0.44 (0.35, 0.55)**
25 to 29	7/318 (2.2)	38/627 (6.1)	77/838 (9.2)	162/902 (18.0)	164/667 (24.6)	REF
30 to 39	11/393 (2.8)	68/966 (7.0)	80/701 (11.4)	121/645 (18.8)	248/822 (30.2)	1.14 (0.96, 1.34)
40 and older	10/785 (1.3)	122/1831 (6.7)	129/1694 (7.6)	214/1548 (13.8)	367/1792 (20.5)	**0.82 (0.7, 0.97)**
Race/ethnicity						
Black, non‐Hispanic	0/60 (0.0)	8/156 (5.1)	13/239 (5.4)	34/294 (11.6)	61/264 (23.1)	1.09 (0.88, 1.37)
Hispanic	3/198 (1.5)	36/602 (6.0)	41/615 (6.7)	81/589 (13.8)	110/675 (16.3)	0.91 (0.76, 1.09)
White, non‐Hispanic	25/1494 (1.7)	184/3003 (6.1)	262/3280 (8.0)	414/3018 (13.7)	642/3161 (20.3)	REF
Other or multiple races	5/155 (3.2)	19/321 (5.9)	30/379 (7.9)	43/366 (11.7)	65/304 (21.4)	1.05 (0.84, 1.32)
Recruitment type						
Gay social networking	2/143 (1.4)	9/167 (5.4)	36/613 (5.9)	65/507 (12.8)	115/592 (19.4)	1.02 (0.84, 1.25)
General gay interest	2/356 (0.6)	4/171 (2.3)	21/176 (11.9)	7/41 (17.1)	12/48 (25.0)	1.37 (0.87, 2.15)
General social networking	7/994 (0.7)	110/2483 (4.4)	153/2348 (6.5)	315/2770 (11.4)	320/1893 (16.9)	REF
Geospatial social networking	22/414 (5.3)	124/1261 (9.8)	136/1376 (9.9)	183/937 (19.5)	443/1939 (22.8)	**1.17 (1.03, 1.33)**
Population density^g^						
Urban	25/861 (2.9)	160/1760 (9.1)	193/1826 (10.6)	378/1829 (20.7)	523/1940 (27.0)	REF
Suburban	2/358 (0.6)	43/760 (5.7)	51/851 (6.0)	80/883 (9.1)	145/901 (16.1)	**0.62 (0.53, 0.74)**
Small medium metropolitan	6/501 (1.2)	39/1180 (3.3)	87/1405 (6.2)	95/1175 (8.1)	186/1274 (14.6)	**0.58 (0.5, 0.67)**
Rural	0/185 (0.0)	5/374 (1.3)	15/422 (3.6)	19/378 (5.0)	37/357 (10.4)	**0.45 (0.33, 0.62)**
Health insurance						
None	0/176 (0.0)	13/287 (4.5)	9/488 (1.8)	62/667 (9.3)	25/399 (6.3)	REF
Private only	10/1040 (1.0)	199/2969 (6.7)	293/3009 (9.7)	432/2723 (15.9)	726/3143 (23.1)	**3.54 (2.40, 5.23)**
Public only	0/90 (0.0)	16/331 (4.8)	25/427 (5.9)	42/380 (11.1)	80/452 (17.7)	**2.98 (1.93, 4.59)**
Other/multiple	3/129 (2.3)	16/288 (5.6)	19/445 (4.3)	33/358 (9.2)	58/358 (16.2)	**2.98 (1.90, 4.68)**
Region						
Northeast	5/392 (1.3)	50/681 (7.3)	62/825 (7.5)	120/810 (14.8)	175/828 (21.1)	
Midwest	10/389 (2.6)	41/855 (4.8)	73/1002 (7.3)	96/812 (11.8)	150/863 (17.4)	
South	7/655 (1.1)	79/1572 (5.0)	112/1630 (6.9)	222/1716 (12.9)	312/1675 (18.6)	
West	11/469 (2.3)	77/966 (8.0)	99/1050 (9.4)	134/927 (14.5)	254/1107 (22.9)	
U.S. dependent areas	0 (0.0)	0/8 (0.0)	0 (0.0)	0/2 (0.0)	0/2 (0.0)	
NHBS city resident						
Yes	18/737 (2.4)	148/1518 (9.7)	167/1601 (10.4)	325/1754 (18.5)	466/1799 (25.9)	
No	15/1170 (1.3)	99/2564 (3.9)	179/2912 (6.1)	247/2513 (9.8)	425/2676 (15.9)	

Chi‐square tests for trend across AMIS cycles in PrEP use overall and within participant subgroups were significant (*p* < 0.05) for all characteristics. A GEE model for linear trend across AMIS cycles in PrEP use overall, controlling for age, race/ethnicity, recruitment source, population density, and health insurance coverage, indicated a significant trend (*p* < 0.0001) overall. aPR, adjusted prevalence ratios; AMIS, American Men's Internet Survey; GEE, generalized estimating equation; MSM, men who sex with men HIV; NHBS, National HIV Behavioral Surveillance; PrEP, pre‐exposure prophylaxis. Bolded values indicate confidedence intervals that exclude 1.0.

a Data collected between December 2013 and May 2014

b data collected between October 2014 and April 2015

c data collected between September 2015 and April 2016

d data collected between September 2016 and February 2017

e data collected between July 2017 and November 2017

f calculated for 2017 sample, using GEE model that adjusted for age (as a categorical variable), race/ethnicity, recruitment source, population density and health insurance coverage

g there were 10 participants in 2013, 11 in 2014, 11 in 2015, 4 in 2016 and 11 in 2017 who reported living in US territories or provided military addresses, which could not have an NCHS urban/rural category assigned.

**Table 5:** EAPC for PrEP awareness, willingness and use among men who have sex with men, by race/ethnicity, 2013 to 2017, United States

	EAPC (95% CI)	
	Non‐Hispanic white	Other race/ethnicity
Aware of PrEP	**12.6 (5.0, 20.8)**	**13.4 (5.4, 22)**
Willing to use PrEP	**3.2 (1.2, 5.3)**	3.0 (−0.04, 6.1)
Used PrEP	**58.8 (41.2, 78.7)**	**66.6 (41.6, 96)**

CI, confidence intervals; EAPC, estimated annual percentage change; PrEP, Pre‐exposure prophylaxis. Bolded values indicate confidedence intervals that exclude 1.0.

Notably, there was also PrEP use reported by men whose responses to behavioural eligibility criteria resulted in their classification as not eligible for PrEP. The proportion of men without a behavioural indication for PrEP who reported PrEP use ranged from 0.5% (2013) to 6.3 (2017; Table 6). When men using PrEP without a behavioural indication were counted as PrEP users and added to the denominator as PrEP‐eligible (e.g. assuming that all men using PrEP had a behavioural indication, even if it was not reported in the survey), the annual prevalences of PrEP use were 2.0% in 2013; 7.5% in 2014; 9.8% in 2015; 17.4% in 2016; 24.3% in 2017.

**Table 6:** Number and proportion of men who have sex with men who reported using PrEP, but whose responses to questions on behavioural indications resulted in their classification as not eligible for PrEP, 2013 to 2017, United States

Survey cycle	Number of men reporting PrEP use who did not report a behavioural indication	Number of men interviewed who did not report a behavioural indication	% of men without a behavioural indication reporting PrEP use
AMIS‐2013	6	1165	0.5
AMIS‐2014	62	3963	1.6
AMIS‐2015	105	4493	2.3
AMIS‐2016	207	4445	4.7
AMIS‐2017	262	4166	6.3

AMIS, American Men's Internet Survey; PrEP, Pre‐exposure prophylaxis.

### 3.4 Trends in usage of PrEP

The proportion of PrEP‐eligible MSM who had used PrEP in the past 12 months increased significantly from 1.7% in AMIS‐2013 to 19.9% in AMIS‐2017 (Table 4). Every racial/ethnic group of MSM had a significant increase in PrEP usage between AMIS‐2013 and AMIS‐2017; for Black MSM, the proportion of PrEP‐eligible MSM increased from 0% in 2013 to 23.1% in 2017. When standardizing by recruitment type across AMIS cycles, non‐Hispanic white MSM had a +58.8% (Figure 1a) EAPC for PrEP usage and MSM of other races/ethnicites had an annual increase of +66.6% of PrEP usage from AMIS‐2013 to AMIS‐2017.

### 3.5 Characteristics associated with PrEP awareness, willingness and use

Using data from the 2017 cycle, compared to MSM aged 25 to 29, MSM aged 40+ were less likely to be aware of PrEP, have used PrEP or be willing to use it (Tables 2, 3, 4). MSM aged 18 to 24 were less likely be aware of PrEP or have used it but were more likely to be willing to use it than those aged 25 to 29. Compared to non‐Hispanic white MSM, all other racial/ethnic groups were less likely to be aware of PrEP. There were no other differences by race/ethnicity. Compared to MSM recruited through general social networking, MSM recruited through geospatial social networking were more likely to have used PrEP and less likely to be aware of PrEP but did not differ in willingness. There were no differences in MSM recruited from general gay interest websites or gay social networking when compared to those from general social networking websites in awareness, willingness or use.

Compared to MSM who living in urban counties, those who lived elsewhere were less likely to be aware of PrEP or have used PrEP (Tables 2, 3, 4). MSM who lived in small/medium metropolitan counties were significantly more likely to be willing to use PrEP than MSM who lived in urban counties. Compared to MSM who had no health insurance, those with any type of health insurance were more likely have used PrEP; however, MSM with any type of health insurance were less likely to be willing to use PrEP. Lastly, compared to MSM with no insurance, MSM with public insurance were less likely to report PrEP awareness. Prevalence analyses and 95% CI for years other than 2017 are not presented.

## 4 Discussion

During a 5‐year period from 2013 to 2017 in the United States, there were substantial, ongoing increases in three key pillars of the PrEP continuum – awareness, willingness and use. These data represent the largest published sample ever used to evaluate the PrEP continuum among MSM in the United States and include data from MSM in rural areas and smaller cities (critical because the use of many prevention services is lower among rural MSM 15), and men recruited through non‐sex‐seeking online venues. Our data extend the findings of the NHBS 16, 17, and previous online studies recruited exclusively from online sex‐seeking websites 18, 19, 20, 21 or in specific US cities 22. Although these data are from a convenience sample and therefore have limited external generalizability, it is critical to triangulate data on PrEP use from multiple sources, in light of their limitations. For example. NHBS data are collected exclusively in urban areas, and data inclusive of urban and rural areas offer an opportunity to compare common outcomes from different non‐representative samples.

The sample of MSM in this study was robust in size and included respondents from all US states. Although the proportionate inclusion of Black (3.1% to 5.7% of PrEP‐eligible) and Hispanic (10.4% to 14.9% of PrEP‐eligible) MSM was lower than their representation in the US population (12% and 17% respectively), participation of Black and Hispanic MSM in AMIS has grown. Regardless, the number of Black (2032) and Hispanic (4957) MSM included in our study make it the largest published survey of PrEP indictors among US Black and Hispanic MSM to date.

The sample of MSM who participated in AMIS was not enrolled with regard to PrEP eligibility. This allowed us to develop an estimate of behavioural PrEP eligibility of 51.4% among MSM in the United States. This estimate is higher than CDC's estimate which suggests that 25% of US MSM are PrEP‐eligible 23. It is not clear whether our estimate or CDC's estimate should be a gold standard. The CDC analysis of PrEP eligibility criteria elements available in NHANES is more limited than those available in AMIS. Also, the NHANES data used for the CDC analysis included data as old as 2007; we have reported recent increasing trends in behavioural risks which might suggest PrEP eligibility for MSM 24. Conversely, our sample might over‐represent men with higher behavioural risks than would be seen in the general population because of our substantial recruitment from geospatial sex‐seeking apps where we have previously reported significantly higher rates of condomless anal sex with partners living with HIV or of unknown HIV status, compared to men recruited from other sources 25.

PrEP awareness increased significantly over the study period. Even so, our report of 47% in 2013 suggests that major increases in PrEP awareness occurred between the time of the initial publication of results of the iPrEX trial in 2010, when two large cohorts of men from sex‐seeking sites 26 and general internet samples 27 reported PrEP awareness of 19% and 29% respectively. It is also important to note that the US Centers for Disease Control and Prevention did not issue the final guidance on PrEP recommendations until mid‐2014; the largest single year increase in PrEP awareness occurred between 2013 and 2014 (the 2014 sample was all surveyed after the publication of CDC's PrEP guidelines). PrEP awareness increased steadily across the period of our study and the rate of increase was comparable between white non‐Hispanic MSM and other MSM.

Willingness to use PrEP grew in the sample overall, and in all demographic groups except MSM of multiple races, men recruited through geospatial social networking apps, and men with certain types of health insurance. The reasons that men who are aware of PrEP are not willing to take it are varied, and have been reported to include concerns about side effects, concerns about drug resistance, low perception of risk for HIV, aversion to daily pill taking, and low perceived efficacy of PrEP 28. Taken together, these data suggest that additional educational efforts could address some of the reasons for unwillingness to take PrEP that are less subjective. For example, educational efforts could be undertaken to increase understanding the side effects are relatively uncommon, and are usually transient 3. Further, the development of drug resistance among MSM taking PrEP has been infrequently reported 29. Although initial reports of PrEP efficacy were modest, subsequent studies, including open label studies, suggest much higher efficacy of PrEP among MSM 3, 30. Although PrEP willingness is increasing, it remains at less than 60% among PrEP‐eligible men in 2017. If we are to achieve 30% to 50% coverage of PrEP, we need to expand willingness in all groups of men, and our data suggest that willingness is lower in men >40 years old and in men with some type of health insurance coverage (compared to men with no health insurance coverage).

PrEP use showed a dramatic increase, from 1.7% of PrEP‐eligible MSM in 2013 to 19.9% in 2017. These increases were observed across all demographic and geographic groups. The annual rate of increase was similar for non‐white MSM and white MSM, although the baseline (2013) prevalence of PrEP use was higher for white MSM than for Black MSM. However, the 2017 levels of PrEP coverage still fall substantially short of the 30% to 50% coverage which is estimated to be required to achieve substantial reductions in population incidence in MSM 4. Although increases in PrEP usage were consistent, there were important differences in the prevalence of PrEP usage by age, recruitment source, population density, and health insurance status. Of special concern, 18‐ to 24‐year‐old MSM in our study, a group that comprised nearly a quarter of all new HIV diagnoses among MSM in 2016 31, have significantly lower PrEP use than their older counterparts. Young Black MSM are at especially higher risk of HIV acquisition, compared to their young white MSM counterparts 32. According to our data, efforts are needed to increase the use of PrEP among at‐risk MSM overall, and specifically among younger MSM, non‐White MSM, among rural MSM, and among MSM without health insurance. Growth in PrEP use might reach saturation among already willing potential users, and any projections of future growth in PrEP use would require an assumption of sustained increases in awareness and willingness. When considering levels of PrEP uptake, it is especially important to recognize the limited PrEP use occurs in a group with high willingness to use PrEP. This suggests that there is disconnect between men's openness to using PrEP and action; this is an important area for research on methods to motivate PrEP uptake and policies that might facilitate men acting on their willingness to use PrEP.

We also report PrEP use among men who did not meet the Public Health Service (PHS) indications for PrEP 33 according to their responses to our behavioural eligibility questions (e.g. evidence of high risk of acquiring HIV). There are several possible explanations for these findings. Men might have had behavioural indications for PrEP more than 12 months before they took the AMIS survey and started PrEP based on those risk indicators but did not have those behaviours during the 12‐month recall period for the survey questions. Alternatively, men might have decided to start PrEP despite not meeting PHS indications for PrEP. Finally, it is possible that men did not report behaviours in their survey responses that they disclosed to providers, or that providers are assessing that men are at high risk for HIV based on criteria other than the PHS criteria. We acknowledge that our estimates of PrEP use among men with PrEP indications are likely under‐estimates of appropriate PrEP use for these reasons.

Our data have limitations that are typical of online surveys. Our sample of MSM was a convenience sample, and does not represent all US MSM, or all internet‐ or app‐using MSM in the United States. Although our sample composition varied from year to year, we had a consistently large sample size, and were able to account for difference in annual sample composition through standardization. Our data are subject to misclassification bias if men did not accurately report their awareness, willingness, or use of PrEP. However, unless that misclassification was differential by year, our conclusions about the trends and direction of changes in our outcomes should be valid. We did not collect data on PrEP persistence, which is a critical threat to the population‐level impact of PrEP on averting new HIV infections 34, 35. The proportion of our respondents who were PrEP‐eligible was 51%, whereas CDC estimates that about 25% of MSM overall are PrEP‐eligible 36. This suggests that some selection bias occurred in our sampling of MSM, resulting in an over‐representation of men at high risk for HIV infection. Future analyses could seek to characterize PrEP use among men with a broader array of specific risk behaviours and combinations of behaviours. Our analysis compared the use of PrEP in different sociodemographic groups of MSM among whom the actual risks of HIV acquisition vary. For example, the risk of HIV infection for Black MSM in the United States is higher than for white MSM because of sexual network and structural factors 32. Thus, although Black and white MSM have similar levels of self‐reported PrEP usage, based on the higher risk of HIV acquisition for Black MSM, the levels of use among Black MSM might still be relatively inadequate. This concept has been formalized as the PrEP to need ratio, which expresses PrEP use in terms of the population‐specific magnitude of new HIV diagnoses 37.

## 5 Conclusions

There has been substantial progress in improving PrEP awareness, willingness and use among MSM in the United States from 2013 to 2017. However, based on modelling results for MSM in the United States 6, 38, PrEP use among US MSM is likely not high enough to produce a substantial 25% reduction in HIV incidence for MSM. To maximize the impact of new PrEP starts on HIV infections averted, new PrEP initiation should be promoted and focused among the highest risk groups of MSM, including Black MSM, Hispanic MSM, and young MSM. In these groups, parity with other race or age groups in prevalence of use or rate of increase is not sufficient for epidemic control or for health equity. Based on the higher risks of infections in these groups and our data showing lower or comparable levels of use in these groups, PrEP coverage targets should be established to have higher relative increases than for other groups of MSM. For Black, Hispanic and young MSM to merely “keep pace” with other race/ethnicity and age groups risks broadening existing disparities in HIV incidence.

Data from our large nationwide annual online survey of MSM provide a valuable tool to monitor the steps of the PrEP continuum in the United States. Routinely collected online survey data are an integral part of a system of indicators of PrEP use, including commercial prescription data and the NHBS. AMIS data supplement these other data sources by providing data annually (important given the very dynamic changes in the indicators from year to year), by providing data from diverse geographic areas in the United States, and by allowing specific indicators for MSM (which are unavailable for commercial prescription data). Our data call for efforts to expand PrEP use among US MSM overall, and especially in among racial/ethnic minority MSM and young MSM, whose risks for HIV acquisition are greater, and among men living in non‐urban areas.

## Competing interests

Dr. Sullivan receives grants and personal fees from Gilead Sciences, outside of this work. The other authors declare no competing interests.

## Authors' contributions

PS conceived of the analysis, directed the analyses, and wrote the paper. TS obtained funding for the data collection, oversaw the study, provided input the analytic plan, and reviewed and approved the final manuscript. MZ implemented the study, conducted the analyses, and reviewed and approved the final manuscript. CC participated in the writing of the manuscript and reviewed and approved the final manuscript. RCS participated in the implementation of the study and reviewed and approved the final manuscript. EK participated in the implementation of the study and reviewed and approved the final manuscript. ST arranged administrative issues and coordinated human subjects approvals, participated in the implementation of the study and reviewed and approved the final manuscript.

## Abbreviations

aPR, adjusted prevalence ratios; AMIS, American Men's Internet Survey; CI, confidence intervals; EAPC, estimated annual percentage change; GEE, Generalized Estimating Equations; MSM, Men who have sex with men; NCHS, National Center for Health Statistics; NHBS, National HIV Behavioral Surveillance System; PHS, Public Health Service; PrEP, Pre‐exposure prophylaxis; STI, sexually transmitted infection.

## Supporting information


**Figure S1.** Characteristics of AMIS 2017 respondents compared to US population, by race/ethnicity, Census region of residence, urbanicity and health insurance status.
