# Supplementary material for: National trends in HIV pre‐exposure prophylaxis awareness, willingness and use among United States men who have sex with men recruited online, 2013 through 2017
Source: J Int AIDS Soc. 2020 Mar 9;23(3):e25461. doi: 10.1002/jia2.25461 (PMC7062633; doi:10.1002/jia2.25461)
Supplement: Supplementary file 1 — Figure S1. Characteristics of AMIS 2017 respondents compared to US population, by race/ethnicity, Census region of residence, urbanicity and health insurance status. [file JIA2-23-e25461-s001.pdf]

Online supplemental Figure: Characteristics of AMIS 2017 respondents compared to US population, by race/ethnicity, Census region of residence, urbanicity and health insurance status.

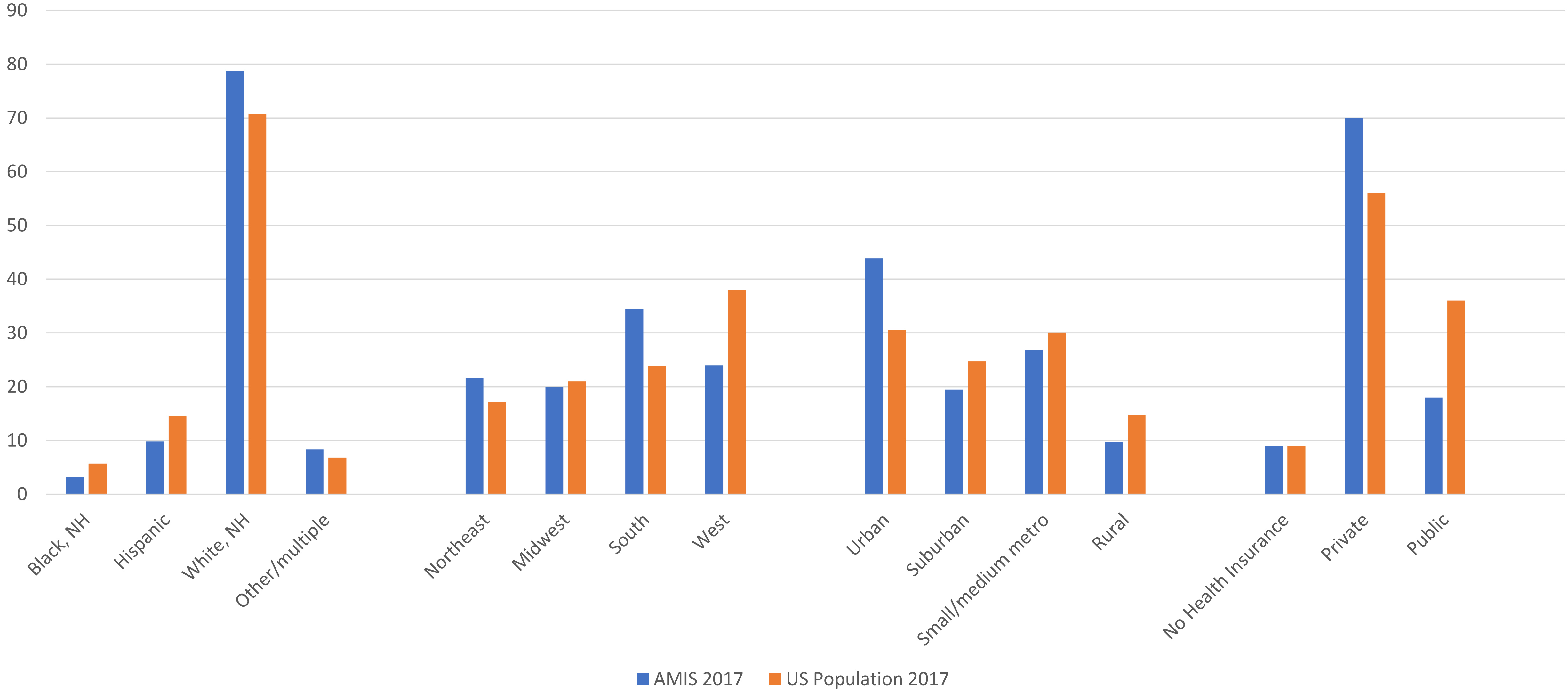

US population Sources: Race: Census FactFinder; Region: https://www.census.gov/popclock/data_tables.php?component=growth; Urbanicity: https://www.cdc.gov/nchs/data/series/sr_02/sr02_166.pdf;
Health insurance: https://www.kff.org/other/state-indicator/total-population/?currentTimeframe=0&sortModel=%7B%22colId%22:%22Location%22,%22sort%22:%22asc%22%7D. Note Kaiser Family Foundation data does not include a category for multiple; based on KFF hierarchy, the AMIS data for other/multiple are categorized as public.
